# Consensus from an expert panel on how to identify and support food insecurity during pregnancy: A modified Delphi study

**DOI:** 10.1186/s12913-022-08587-x

**Published:** 2022-10-05

**Authors:** Fiona H. McKay, Julia Zinga, Paige van der Pligt

**Affiliations:** 1 School of Health and Social Development, Institute for Health Transformation, Faculty of Health, Deakin University, 3220 Geelong, VIC Australia; 2 Department of Nutrition and Dietetics, Royal Women’s Hospital, Parkville, VIC Australia; 3 The Institute for Physical Activity and Nutrition (IPAN), School of Exercise and Nutrition Sciences, Faculty of Health, Deakin University, 3220 Geelong, VIC Australia; 4 Department of Nutrition Western Health, Footscray, Australia

**Keywords:** Food security, Pregnancy, Delphi, Practice guidelines

## Abstract

**Background:**

Food insecurity and hunger during pregnancy have significant implications for the health of the mother and baby. Assisting clinicians when they encounter women who are experiencing hunger or food insecurity during their pregnancy will increase the opportunity for better birth and pregnancy outcomes. At present there are no guidelines for Australian clinicians on how to do this.

**Methods:**

This study uses a modified Delphi technique, allowing diverse participation in the process, to create consensus on the ways to address and respond to food insecurity during pregnancy. This modified Delphi collected data via two rounds of consensus. The opinions collected from the first round were thematically categorised and grouped. The topics were integrated into the survey for the second round and circulated to participants. During the second round, priorities were scored by giving five points to the topic considered most important, and one point to the least important.

**Results:**

Through two rounds of consultation, the panel achieved consensus on how to identify food insecurity during pregnancy, with some clear items of consensus related to interventions that could be implemented to address food insecurity during pregnancy. Experts achieved consensus on items that have importance at the institution and policy level, as well as services that exist in the community. The consensus across the spectrum of opportunities for assistance, from the clinical, to community-provided assistance, and on to government policy and practice demonstrate the complexity of this issue, and the multipronged approach that will be required to address it.

**Conclusion:**

This is the first time such a consultation with experts on hunger and food insecurity during pregnancy has been conducted in Australia. Items that achieved consensus and the importance of the issue suggest several ways forward when working with pregnant women who are hungry and/or food insecure.

**Supplementary information:**

The online version contains supplementary material available at 10.1186/s12913-022-08587-x.

## Introduction

Food insecurity, defined as inadequate access to healthy, affordable, and culturally appropriate food, impacts more women than men, particularly those of reproductive age [1, 2]. Food insecurity and hunger during pregnancy have significant implications for the health of the mother and baby. Pregnant women who are food insecure frequently have poor diet quality and sub-optimal nutritional intake during pregnancy, leading to negative maternal and child health outcomes [3, 4]. When compared to women who are food secure, women who are food insecure during pregnancy are at a higher risk of gestational diabetes [5], low birth weight [6, 7], maternal stress [4, 8], excess maternal weight gain [5], birth defects [9], premature birth, and struggle to breastfeed [10, 11]. The impacts of food insecurity, and as a result inadequate nutrition, during pregnancy can be both significant and long term for mother and child, leading to challenges with child growth and development [4, 5, 12, 13].

Approximately 1 in 10 pregnant women are food insecure in Australia [14]. A recent systematic review of interventions specifically focused on addressing food insecurity during pregnancy found that the main interventions are nutritional supplementation and/or nutrition education, [15] however, the limited number of robust evaluations or long-term interventions mean that evidence for any one intervention type is limited. Recent research has found that while health care providers in one Australian antenatal setting were aware of the importance of maternal nutrition for the short- and long-term health of both the mother and baby, they were uncertain how to broach issues surrounding food insecurity, and when they do, they have few strategies to assist the hungry or food insecure parent [16]. Assisting clinicians when they encounter women who are experiencing hunger or food insecurity during their pregnancy will increase the opportunity for better birth and pregnancy outcomes [17]. Clinical practice guidelines are often used in these situations to provide guidance for clinicians when dealing with patient concerns, however, as yet there are no such Australian guidelines or advice that assist antenatal management of women who are food insecure both during and following pregnancy.

The development of clinical practice guidelines and other forms of clinical advice traditionally consists of gathering scientific evidence, and formal and explicit consensus judgement methods [18]. This study uses a modified Delphi technique, allowing diverse participation in the process, to create consensus on the ways to address and respond to food insecurity during pregnancy with the information able to be used for the development of clinical practice guidelines for use by antenatal clinicians to assess and respond to food insecurity and hunger during pregnancy [19–21].

## Method

### Study design

This study employed a modified Delphi approach to gain consensus and seek expert opinion in an iterative structured manner [19–21]. The Delphi approach involves key stakeholders and experts, and often uses focus groups or individual interviews, workshops, meetings, or seminars [22, 23]. However, these methods typically require face-to-face interaction, a challenge during COVID-19 related travel limitations, and a process that does not allow for the engagement of people from disparate geographical regions. To overcome these challenges, this study employed a modified Delphi approach where consensus was achieved via online methods. Such an approach has been said to be characterized by greater openness attributed to anonymous participation [24], and an increased diversity of participants thanks to the increased accessibility of the online format [25]. Due to the exploratory nature of this work, this modified Delphi also included open-ended questions to gain further insight from experts. The use of open-ended questions allows items to be generated by the expert panel organically, in addition to the items that are generated and included from a review of the literature [26].

There are a number of guidelines for using the Delphi approach to achieve consensus [22]. This study defines consensus as 75% agreement combined with results of ranking, a suggestion made by both Diamond, Grant [22] and Foth, Efstathiou [27] who highlight the importance of defining consensus prior to beginning the first round. The combination of these two techniques, percentage agreement and ranking, allowed for consensus to be achieved in two rounds. The Delphi procedure allows for flexibility in delivery and number of rounds, typical modifications restricting the number of rounds to two or three due to the difficulty of sustaining a high response rate over subsequent rounds [28–30]. Including two rounds is supported by a large body of research that employs the Delphi procedure [31–33]. Kaynak and Macaulay [34], suggest that rather than being employed as a decision-making tool, the Delphi technique should be considered a tool of analysis. This means that the aim is not to achieve a definitive answer, but instead, to aid in the development of possible solutions. As a result, it is not necessary to continue rounds until all items reach consensus, but only until a clear pattern is discerned. In the current study, round one consisted of suggestions for practice based on current evidence [15] and asked participants to make suggestions on actions they considered useful in responding to food insecurity and hunger during pregnancy. The inclusion of open-ended question in the first round is consistent with previous research suggesting that the first round be as exploratory as possible [35], the subsequent round followed, where these suggestions were ranked and refined, a common feature of Delphi approaches as described in the literature [23, 36, 37].

## Participants and recruitment

There is little agreement on the number of participants required to achieve consensus [23]. Linstone and Turoff [19] recommend a minimum of ten participants, acknowledging that when increased beyond this number, the Delphi can become labour intensive, with a large amount of data being gathered. While Okoli and Pawlowski [38] suggest a sample of between 10 and 18 sufficient to achieve consensus. Furthermore, it has been reported that improvements in reliability once the number of experts in the panel rises above 15 are negligible [39]. As a result of the level of time commitment required from the panel members, Hanafin and Brooks [40] suggest attrition rates of 16–28% should be expected per round. Allowing for this level of attrition and aiming to have at least ten contributors in the second round, we aimed to recruit a sample of 15–20 experts. While there is little agreement on the size of the sample [39], critical in the membership of the panel is that it is balanced in terms of the composition of members from different areas of expertise and experience [41, 42].

Participants were recruited through professional networks of the researchers via email, direct contact through publicly available contact information, and via social media (Twitter and LinkedIn) to reach a broad audience. Participants were also invited to share the invitation to the first round with people in their network who might also have expertise in nutrition, pregnancy, and food insecurity. The recruitment material included a link where potential participants could read the plain language and informed consent statement and provide their contact details.

## Data collection

Those who expressed their interest in being involved in the study through the recruitment procedure described above were provided with a link to an online survey via email. This modified Delphi collected data via two rounds of consensus.

**Round 1:** In round one, participants were asked to identify possible interventions which might be effective for targeting food insecurity among pregnant women, and considerations when dealing with food insecurity among pregnant women. They were asked to rank how important food insecurity is during pregnancy, whose responsibility it is to manage and respond to food insecurity, and if consideration of food insecurity should be included in standard clinical practice. In addition, experts were asked to suggest at least five potential aspects of care that need to be considered when supporting a pregnant woman who was food insecure, at least five possible ways to address food insecurity, and at least five possible barriers to addressing food insecurity among pregnant women. These suggestions were thematically collated for rating and ordering in the subsequent round. Participants were also asked to identify their main areas of professional practice, where they are allocated, and to self-identify their level of expertise from 1 (novice or in training) to 10 (expert). The round one survey was open for four weeks to include as many experts as possible.

**Round 2:** Participants who completed round one were sent a summary via email of the current research that seeks to address food insecurity in pregnancy. This summary was based on a systematic review [15] completed by the authors and describes the current situation of food insecure pregnant women and current evidence-based interventions. The summary of previous evidence was provided in round two, rather than earlier, to discourage the bandwagon effect [43], a common limitation of Delphi, allowing for free flowing ideas to be generated in round one. This material was designed to orientate the expert to the focus of the study, a process that has been found to be a useful way to build the research relationship and provide the experts with an easy summary of the current evidence [44].

A list of all suggested priorities and ways to respond to food insecurity during pregnancy, based on the outcomes of round one and the current literature, were compiled into a new survey and emailed to each expert who was involved in round one. Experts were asked to rate each item in terms of importance on a 5-point Likert scale (1 = not important at all, 2 = not very important, 3 = moderately important, 4 = very important, 5 = extremely important). A free‐text space was provided for feedback on the items and to comment on their decision-making process. The experts were given two weeks to complete their responses and were sent a reminder via email after the two weeks had lapsed.

### Data analysis

The opinions collected from the first round were thematically categorised and grouped. The topics were integrated into the survey for the second round and circulated to participants. During the second round, priorities were scored by giving five points to the topic considered most important, and one point to the least important, the mean score for each item was calculated. Responses for extremely important and very important were groups together to determine consensus. Participants were asked to choose five of the same items to be ranked from highest to lowest priority. Participants were given the opportunity to provide qualitative responses related to their selection, these were thematically analysed and are presented here verbatim [45].

## Results

In total, 12 experts completed round one of the Delphi and 11 completed round two. The one participant who completed round one but not round two was followed up via email twice, but made no reply. Participants were located in various states in Australia; four in Queensland, three in Tasmania, two in Victoria, and one each in New South Wales, and the Australian Capital Territory. Participants had expertise in academia and/or research (n = 6) and in clinical practice as a midwife or dietitian (n = 9); most (n = 9) identified themselves as having a high level of expertise with food insecurity, hunger, and pregnancy. See table one for demographic characteristics of the sample (Table 1).

**Table 1﻿:** Demographics of the final sample (n = 11)

Sex	
Female	11 (100)
**State of professional practice**	
Queensland Tasmania Victoria New South Wales Australian Capital Territory	4 (36) 3 (27) 2 (18) 1 (9) 1 (9)
**Self-reported Level of expertise**	
0–3 Novice 4–6 In training 7–8 High level of experience 8–10 Expert	1 (9) 1 (9) 5 (45) 4 (36)
**Main area of expertise** ^*^	
Dietitian and nutritionist Research/academic Midwife Government	7 (63) 5 (45) 2 (18) 1 (9)

^*^ Participants were able to choose more than one option, percentages do not total 100

Most participants (n = 10, 83%) considered food insecurity to be a serious concern for mother and baby during pregnancy. These concerns related to participants concern about the consequences of food insecurity during pregnancy for both mother and baby.

If a woman is food insecure it will compromise short- and long-term outcomes for her and her offspring and intergenerationally (Research/academia, Dietitian/nutritionist, expert).

With those participants who were also clinicians were concerned about both the physical impacts of food insecurity and hunger and about the mental health implications, both in the short and the long term.

Immediate risk for nutritional deficiency for key nutrients e.g., iron, folate, iodine. There is also the stress of being able to feed oneself and support a healthy pregnancy. Long term affects relationship with food and eating behaviours which can influence both their own health and their feeding of their child. Can set up disordered eating (Dietitian/nutritionist, expert).

Concerns related to the multiple factors that can impact food insecurity were acknowledged by other participants. These factors were said to be exacerbated by the COVID-19 pandemic as financial security during periods of restrictions impacted people’s employment and food security.

Through my work in Tas (Tasmania) I have learnt there can be many different factors responsible for food insecurity during pregnancy and post-partum eg domestic violence, job loss, if people are struggling there are major hoops to jump through to get support payment which wouldn’t go very far at all. The pandemic has not helped in this area either (Dietitian/nutritionist, high level of experience).

Most participants suggested (n = 11) that asking women about their food security status pregnancy should be standard part of clinical practice. Participants suggested that this should be included in general practice and pregnancy care.

Should be more included in pregnancy care guidelines to raise the importance of this area, care should be provided in a team approach with food and eating advice, access to food relief and other social supports. There needs to be more investment and critical looking into the system. Access to income support payments in pregnancy do not reflect the increased need at this time putting the health of pregnancy at risk (Dietitian/nutritionist, high level of experience).

Others considered this to be a ‘system’ problem, with the real solution lying in system change spearheaded by governments, and in the absence of governments, an approach that brings in other actors within the healthcare system to provide comprehensive care to people who are food insecure.

The government – policies should exist that eliminate food insecurity. Until they do so, a multidisciplinary approach is most appropriate – primary care, social work, mental health, dietitians (Midwife, expert).

Participants were asked to identify barriers that prevent them from addressing food insecurity among their clients. These barriers can be grouped into three broad categories. The first are those barriers that mean a clinician cannot personally assist a patient or client, for example, some suggested that there was insufficient education about food insecurity in the midwifery curriculum, while others highlighted the time barriers they face when providing care. The second barrier relates to a misunderstanding or uncertainty about whose responsibility addressing food insecurity among patients or clients is, or about how important addressing food insecurity is when there may be other concerns, including those specifically related to pregnancy, or others such as domestic violence, mental illness, or homelessness. The final barrier relates to the systemic level challenges that prevent participants from providing assistance related to food insecurity to clients or patients. System level barriers include a lack of government financial support, to those that exist at the health care level, including timing of provision of care and a lack of routine food insecurity screening.

## Identification of a food insecure pregnant woman

Participants were asked to rate the importance of a range of considerations when supporting or identifying a pregnant woman who is (or who they suspect to be) food insecure or hungry (Table 2). Of the nine statements posed, eight achieved consensus, with five achieving 100% agreement. Mean scores for eight of the nine statements were over 4 out of a possible 5. Agreement and rank prioritisation were consistent. Participants identified linking women with appropriate social care services such as emergency community food assistance as both the most important consideration and as the highest priority. The item that was ranked as the lowest priority and where only 64% of participants indicated that this was extremely or very important was providing women with food literacy or nutrition education.

**Table 2:** Considerations when identifying a food insecure pregnant woman

	Percentage agreement	Mean	Rank
Linking women to appropriate social care services	100	4.9	1st
Assessing domestic or family violence	100	4.5	2nd
Linking women to income support services	100	4.6	3rd
Linking women to appropriate health care services	100	4.7	4th
Assessing access to nutrition supplements	82	4.1	5th
Considering the culture and/or the family unit	100	4.5	6th
Determining if there are other health conditions to consider	91	4.5	7th
Assessing previous experience with food insecurity	91	4.2	8th
Providing food literacy or nutrition education	64	3.7	9th

## Addressing food insecurity during pregnancy

Participants were asked to rate the importance of a range of actions when addressing food insecurity during pregnancy (Table 3). Of the 14 statements posed, ten achieved consensus, with one achieving 100% agreement and seven achieving 91% agreement. All the actions that achieved consensus were system level actions, that could be achieved through policy or institution level cooperation. Agreement and rank prioritisation were consistent. Mean scores for 10 of the 14 statements were above 4 out of a possible 5. Participants identified creating a social care arrangement specifically for food insecure pregnant women, one that might include access to nutrition supplements, and care and support for pregnant women that focuses on reducing stigma and blame, as the key priority and the most important action. Items that were ranked as the lowest priorities included providing women with food literacy or nutrition education, linking women with other health care services, and directly providing food (either through food parcels or via commercial meal kits).

**Table 3:** Actions when addressing food insecurity during pregnancy

	Percentage agreement	Mean	Rank
Create specific social care services for pregnant women	100	4.8	1st
Refer women to emergency and community food assistance	91	4.5	1st
Advocate for adequate income support	91	4.7	2nd
Routine food security screening	91	4.7	2nd
Government funded food security program	82	4.3	3rd
Linking women to appropriate social care services	91	4.4	3rd
Reducing stigma surrounding asking for help for food	91	4.7	4th
Provide cash vouchers for food	73	4.2	5th
Advocate for stable and affordable housing	91	4.5	6th
Linking women to income support services	91	4.4	7th
Provide nutritional educational and literacy programs	45	3.5	7th
Linking women to appropriate health care services	55	3.8	8th
Provide food parcel	64	3.9	8th
Provide commercial meal kits	40	3.2	9th

## Interventions to address food insecurity during pregnancy

Participants were asked to rate the importance of a range of activities that could be implemented to address food insecurity during pregnancy (Table 4). Of the nine statements posed, four actions achieved consensus, two achieved 100% agreement, one was determined to be approaching consensus (73% agreement). Mean scores for five of the nine statements were above 4 out of a possible 5. Actions that were highly ranked were those which can be influenced at the clinic level, including routine food security screening, the introduction of clinical practice guidelines, and referral to emergency and community food assistance; these items are consistent with other questions in round two and results of round one. Items that were ranked as the lowest priorities included providing women with food literacy or nutrition education, and directly providing food (either through food parcels or via commercial meal kits).

**Table 4:** Interventions that could be implemented to address food insecurity during pregnancy

	Percentage agreement	Mean	Rank
Routine food security screening	100	4.9	1st
Clinical practice guidelines	73	4.3	2nd
Comprehensive food security program	82	4.3	3rd
Referral to emergency and community food assistance	100	4.5	4th
Cash vouchers for food	64	3.8	5th
Universal basic income	82	4.5	6th
Nutritional educational and literacy programs	55	3.7	7th
Food parcel	55	3.6	8th
Provide commercial meal kids	36	3.1	9th

## Discussion

This study adopted a modified Delphi to facilitate a systematic and rigorous consultation exercise on the issue of food insecurity and hunger during pregnancy. This study drew on the clinical and research experience of an expert panel on food insecurity, hunger, and pregnancy in Australia. Through two rounds of consultation, the panel achieved consensus on how to identify food insecurity during pregnancy, with some clear items of consensus related to interventions that could be implemented to address food insecurity during pregnancy. This is the first time such a consultation with experts on hunger and food insecurity during pregnancy has been conducted in Australia, the items that achieved consensus and the importance of the issue suggest several ways forward when working with pregnant women who are hungry and/or food insecure.

Experts achieved consensus on items that have importance at the institution and policy level, as well as services that exist in the community. The consensus across the spectrum of opportunities for assistance, from a clinical or institutional level, to community-provided assistance, and on to government policy and practice demonstrate the complexity of this issue, and the multipronged approach that will be required to address it. Of importance when considering these responses is that experts in this study were not looking for a band aid or temporary solution to issues of food insecurity and hunger during pregnancy, but, rather, were seeking a solution that was both tested and found effective and addressed some of the structural reasons that people are food insecure.

## Institutional level solutions

Items that achieved consensus that could be implemented at the institution level, for example at individual clinics or hospitals or indeed in all hospitals, include routine screening and linking pregnant women with a range of services, both internal and external to the clinical setting. Including routine screening and then providing programs that link food insecure households or individuals to the services via health care settings is a solution that has grown out of the suggestion that supporting food security can lead to improvements in population health [46, 47]. There is increasing interest in routine screening and the role of healthcare systems in addressing food insecurity in non-pregnancy healthcare settings [48, 49]. The International Classification of Diseases, Tenth Revision, Clinical Modification (ICD-10-CM) Z codes (Z55–Z65) allow for the classification and documentation of the social determinants of health in electronic medical health records. The official guidelines for coding and reporting of the ICD-10-CM suggest that all clinicians, not just physicians involved in the care of a patient, document the Z codes to report on the social determinants of health of the patient [50]. Screening tools that are based on or match these codes could be incorporated into existing screening mechanisms or could be used as the basis a more comprehensive referral system.

While screening for food insecurity does not exist in the Australian health care setting, screening exists in health care settings in other countries, where it has been found to be part of a successful approach when seeking to address food insecurity [51–53]. Both the American Academy of Family Physicians, and the American Academy of Paediatrics recommend that food insecurity is routinely screened [54]. A systematic review of routine screening in the health care setting found that screening is generally conducted via a brief screening tool comprising one or two items [54]. As the results of this current study demonstrate, there is an appetite for routine screening from clinicians to identify and assist food insecure pregnant women. In Australia, there is work underway that seeks to highlight the link between health care settings and actions that address food insecurity. For example Kerz, Bell [48], have validated a brief tool to be used in a Australian paediatric health care setting, with findings suggesting that food insecurity is prevalent among families of children attending paediatric outpatient hospital appointments. While McKay and colleagues [14] have demonstrated that a brief tool measuring food insecurity could be used in a clinical setting as a component of a referral pathway for pregnant women who are identified as food insecure. It is possible that this two-item tool could be included in the electronical medical health record and incorporated into standard practice. While there is evidence for the importance and acceptability of screening for food insecurity, practical considerations need to be made. For this reason, researchers suggest that short screening measures be employed [14, 48]. Short screening measures are limited in that they do not allow for the assessment of the severity of food insecurity, however, they are more appropriate for busy clinical settings and take into consideration barriers including time constraints and increased workloads experienced by clinicians, while still being able to determine food secure status [55]. While it is clear that clinicians consider food insecurity screening as an important component of antenatal healthcare [16, 56], there are a variety of barriers that may prevent them from asking their patients about their household food security. This includes a lack of guidelines, uncertainty surrounding responsibility for screening, inadequate clinical knowledge and training, and time constraints [16, 56].

While approaches to screening are gaining traction, there remain gaps in how to best link food insecure pregnant women to the services they need. A recent systematic review exploring interventions that have sought to address social needs during pregnancy care after standard or routine screening, found that while there are evidence based interventions for family and domestic violence, there are few interventions for other social needs, including for people who have been identified as food insecure [57]. A different review found that despite an increase in the number of care settings that screen for food insecurity, those who are referring food insecure or hungry women and families, are largely referring them to external services (for example, in the USA supplemental nutrition assistance program, SNAP, and women, infants, and children supplemental nutrition assistance program, WIC, are common places of referral). However, referral and assistance can be less formal and include providing information about emergency and community food assistance, as well as providing assistance via community health workers or social workers [58]. Research suggests that these linkage programs have positive health outcomes [59].

## Community level solutions

Experts rated community level solutions, such as linking women to appropriate income support and social care services, and referrals to intimate partner violence services. The consideration of these community levels services reflects an acknowledgement that while food insecurity has clinical implications, the solutions may lie outside the hospital setting, with structural and systemic level changes known to take considerable time, highlighting the need for immediate solutions to solve short term need. While the definition of food insecurity is a lack of appropriate food, there are many reasons that individuals and households are unable to access food. Estimates suggest that 13% of Australian’s live below the poverty line [60] due to rising living costs, stagnant wage growth, and unemployment and underemployment. Poverty predisposes low-income individuals towards a suboptimal diet [61]. Many people who live below the poverty line are also in receipt of government welfare payments, however, these payments may be insufficient to cover the basic costs of living, increasing stress and pressure on individuals and households. There is a body of research that highlights the role of poverty and income on chronic food insecurity [62], with many people who live on low incomes forgoing food for other basic living expenses [63]. Low-income neighbourhoods are more likely to have limited options for fresh produce and whole grains [64], and are more likely to have access to fast-food outlets and convenience stores [65]. Low income households are often forced to make decisions about the food they can purchase, sometimes in the absence of health considerations [66]. For many individuals and families, the experience of food insecurity and its impact on diet is greater than limited funds and lack of access to healthy food, and while many households experience short term or acute food insecurity, for other families, the experience of food insecurity can be long term, often intergenerational [67, 68]. While there is evidence to suggest that at current levels, government-provided income support is below the poverty line for most families, it can provide some mitigation from the more serious impacts of food insecurity [69, 70] and as highlighted by experts in the current study, referral to appropriate income support should be included in any approach to address household food insecurity in a health care setting.

In addition to the physical experience of hunger and the physiological impacts of poor nutrition, there are also psychological implications for food insecurity. Work from the USA has identified a relationship between receipt of welfare and intimate partner violence, finding that intimate partner violence was associated with negative health outcomes and greater material hardship [71]. There is a significant amount of evidence highlighting the risk of domestic violence during pregnancy [72], and there is emerging evidence suggesting a relationship between food insecurity during pregnancy and intimate partner violence. According to Ricks, Cochran [73], food insecurity can be linked to violence in three main ways. First, economic abuse can produce food insecurity, as one partner in the relationship controls or restricting access to finances by the other partner [74]. Second, many individuals who escape an abusive relationship rely on financial assistance and low-wage jobs for survival, therefore lacking the financial ability to secure food. Third, some evidence to suggest that a food insecure environment may increase the rate of violence [75, 76]. The relationship between food insecurity, pregnancy, and intimate partner violence is unidirectional. Pregnant women who are food insecure are more likely to experience violence from an intimate partner [76, 77], and there is a predictive effect of intimate partner violence on food insecurity in longitudinal studies [78]. There are a range of factors for intimate partner violence, including drug and alcohol use, prior violence, traditional attitudes to gender and a range of socio-demographic characteristics [79]. As highlighted by experts in this study, there is a need to consider intimate partner violence when considering food insecurity. Screening for intimate partner violence already exists in most antenatal care in Australia, and as such most practitioners working in this space will already be able to screen for intimate partner violence, posing an opportunity to include food insecurity screening at the same time [80]. Promisingly, a recent systematic review suggests that pregnant women who are experiencing intimate partner violence with or without other mitigating factors are likely to benefit from screening, referral, and supportive counselling [81].

The main community level responses to food insecurity and hunger in Australia are through emergency and community food assistance; foodbanks and pantries, soup kitchens, and school lunch and breakfast programs [68]. While these community solutions play an important role in the charitable response to food insecurity and hunger, many people who use these services experience shame and stigma [82], and various restrictions on how and when they can be used means they are generally not able to meet all the needs of those who are experiencing hunger and food insecurity [83]. While many Australian emergency and community food assistance providers refer clients on to other services including family violence and income support services [84], to date, there has been limited evidence to suggest that clinicians refer patients to emergency and food assistance providers, nor that this is something that they would consider doing [16]. Unlike the more formal system in the USA, in Australia, these services are typically informal and are not a part of a systemic approach to food insecurity through partnership with government, health care, and charity. Encouraging research from the USA highlights a growing body of evidence demonstrating positive partnerships between healthcare systems and local food assistance programs as a way to reduce food insecurity and hunger and assist people to access the services they need [58, 85]. This research suggests that such a partnership may result in increased food intake, including increased fruit and vegetable intake [86], and better health outcomes [87, 88].

## Government level solutions

While most high-income countries have some form of government assistance for those who are experiencing hunger or food insecurity, these programs can vary widely. As described above, the main response of governments to food insecurity and hunger in Australia is through emergency and community food assistance. This sector has grown since the 1990s, most rapidly in the past decade. The sector in Australia operates through both formal and informal networks and is comprised of food banks, food pantries, soup kitchens, and meals programs operated by charities [89]. Foodbank Australia, Australia’s largest re-distributor of community food, has reported an increase in the number of people accessing their services, with almost one million people receiving food from Foodbank each month in 2021 [90]. This system is complemented by a range of government-provided or -run welfare programs that provide income support for aged, disabled, parents of young children, people seeking employment, and family tax benefits that help low-income families with the cost of raising children. Unlike the system in the USA, there is no large government program that is specifically designed to provide food assistance. The USA in comparison has multiple federal programs that are specific to food aid. The main federal food assistance programs include the Supplemental Nutrition Assistance Program (SNAP); the Special Supplemental Nutrition Program for Women, Infants, and Children (WIC); and the National School Lunch Program (breakfast, lunch, and summer meals) [91].

Experts identified structural solutions to food security as possible ways to address food insecurity during pregnancy and as interventions that could be tried. While Australia does not currently have a comprehensive food security program like that of the USA, we may have reached a time when key stakeholders need to advocate for one. Enrolment in federal food assistance programs in the USA is associated with improved outcomes across multiple dimensions, including food security, nutrition, health, development, and health care costs [92, 93]. Importantly, the WIC program has been found to significantly reduce food and nutritional security among both pregnant women and children [94, 95]. Such a program allows for a clear referral pathway for women who are identified as food insecure when pregnant and ensures a systematic approach to ensuring adequate food and nutrition [52, 96].

## Limitations

While it is clear that solutions to targeting food insecurity in pregnancy have been acknowledged by experts in this area, there are a number of limitations that need to be taken into consideration. The aim of the study was to include 15–20 experts in round one in order to maintain the 10 needed for round two. Despite a very board recruitment campaign, this was not achieved, and more participants may have provided a diversity of opinions. However, most participants (11 of 12) who completed round one responded to round two, thereby eliminating the problem of attrition common in other Delphi studies. Secondly, COVID-19, and time and financial constraints meant that a face-to-face Delphi was unachievable. We chose a modified Delphi to attract a broad range of experts from all over Australia. Having an in-person meeting may have altered the results or provided more solutions. This study does not include the voices of consumers, and as such, solutions that may have been identified by those experiencing food insecurity may have been missed. However, a recent Australian study with pregnant women and their experience of hunger and food insecurity suggested that that while over half (57.1%) were comfortable that their health care provider might asked them about their household food security, most (61.6%) did not expect to be asked [97]. Finally, as described all participants were clinicians and were not experienced in policy or in working at a system level, this could be a limitation in both the suggestions provided here and the possible outcomes of this work if taken up by clinicians. Despite these limitations, the consensus in this study is a strength and provides support for the results presented here.

## Conclusion

Through a rigorous and systematic modified Delphi, we have been able to provide a number of suggestions, supported by a panel of experts, on how to identify and support food insecure pregnant women in a clinical setting. The logical next step from this study is the creation and testing of acceptability of clinical practice guidelines for the assessment and support of food insecurity during pregnancy. This is a significant gap in clinical care in the Australian health care setting.

## Electronic supplementary material

Below is the link to the electronic supplementary material.

Supplementary Material 1

## Data Availability

The datasets generated during and/or analysed during the current study are not publicly available but are available from the corresponding author on reasonable request.
