# Supplementary material for: Consensus from an expert panel on how to identify and support food insecurity during pregnancy: A modified Delphi study
Source: BMC Health Serv Res. 2022 Oct 5;22:1231. doi: 10.1186/s12913-022-08587-x (PMC9533284; doi:10.1186/s12913-022-08587-x)
Supplement: Supplementary file 1 — Supplementary Material 1 [file 12913_2022_8587_MOESM1_ESM.pdf]

Delphi

Start of Block: Default Question Block

Q1
Researchers at Deakin University are conducting a Delphi to determine consensus on how to address food insecurity during pregnancy.

We are conducing a three round Delphi, this is round one. Round one of this survey will take around 15-20 minutes.

Please click here for more details about the study before proceeding: Participant Information & Consent Form

Do you consent to participating in this study by completing this survey?

- Yes (4)
- No (5)

Page Break

Q4 Please provide your details below.
The information will be used only to identify participants for round 2 of the survey.

- ○ Name (1) ____
- ○ Employer (2) ____
- ○ Email address (3) ____

Q3 Please identify your main areas of expertise so that we can understand the profile of responders contributing to this consultation.
Please choose the options that apply to you

- ▢ Research/academia (4)
- ▢ Dietitian and nutritionist (5)
- ▢ Midwife (6)
- ▢ Obstetrician (7)
- ▢ Other (8) ____

Q2 Where are you located (for the purposes of your expertise)?

- ACT (1)
- NSW (2)
- QLD (3)
- VIC (4)
- WA (5)
- Tas (6)
- NT (7)
- SA (8)

Q15 How would you rate your level of expertise?

	Novice or training							Expert			
	0	1	2	3	4	5	6	7	8	9	10

Page Break

Q6 These first questions are designed to seek your personal opinion

---

Q5 How serious of a concern do you think that food insecurity is for pregnancy (for mother and/or baby)

- ○ Extremely bad (1)
- ○ Somewhat bad (2)
- ○ Neither good nor bad (3)
- ○ Somewhat good (4)
- ○ Extremely good (5)

Q9 Why did you respond the way you did to the above question?

____

Q7 Do you think asking questions about food insecurity should be included in standard clinical practice for pregnant women?

- ○ No (1)
- ○ Yes (2)
- ○ Unsure (3) ____

Q8 Who do you think should be responsible for addressing food insecurity among pregnant women?____

Page Break

Q11 Consider the following statements and determine how much you agree or disagree.

Q10 Food insecurity status should be determined for all pregnant women

- ○ Strongly disagree (1)
- ○ Somewhat disagree (2)
- ○ Somewhat agree (3)
- ○ Strongly agree (4)

Q12 Clinical staff play a role in addressing food insecurity among pregnant women

- ○ Strongly disagree (1)
- ○ Somewhat disagree (2)
- ○ Somewhat agree (3)
- ○ Strongly agree (4)

Q13 Food insecurity should be considered like any other medical condition when attending a pregnant women

- ○ Strongly disagree (1)
- ○ Somewhat disagree (2)
- ○ Somewhat agree (3)
- ○ Strongly agree (4)

Q14 In no particular order, please list 5 things that you think should be considered when dealing with a pregnant women who is (or who you suspect to be) food insecure or hungry.

- ○ 1 (1) ____
- ○ 2 (2) ____
- ○ 3 (3) ____
- ○ 4 (4) ____
- ○ 5 (5) ____

Q19 Please comment on why you think these are important

____

Q18 In no particular order, please list 5 ways that you think we could address food insecurity or hunger during pregnancy.

- ○ 1 (1) ____
- ○ 2 (2) ____
- ○ 3 (3) ____
- ○ 4 (4) ____
- ○ 5 (5) ____

Q20 Please comment on why you think these are important

____

Q16 In no particular order, please list 5 things that might prevent addressing food insecurity and hunger during pregnancy

- 1 (1) ____
- 2 (2) ____
- 3 (3) ____
- 4 (4) ____
- 5 (5) ____

Q17 Thinking about the 5 barriers above, what ways might we address these barriers, either in a clinical or non clinical space

____

Q21 In an ideal world, how would you want to address food insecurity and hunger during pregnancy, are there any interventions or programs that you know of or that you would like to try?

____

Q22 Thank you!

This first stage of the Delphi survey is now complete.

In approximately three weeks you will receive the second stage of the survey which will anonymously summarise all the responses we received and ask some further questions as we together try to build consensus on ways that we can address food insecurity during pregnancy.

Feel free to contact Fiona McKay directly if you have any questions (fiona.mckay@deakin.edu.au).
If you have any further comments or questions about the Delphi survey overall please provide them here:____

End of Block: Default Question Block
